# A Coaching-Based Leadership Program for Women Postdoctoral Fellows at the National Cancer Institute that Cultivates Self-confidence and Persistence in STEMM

**DOI:** 10.1007/s13187-024-02466-1

**Published:** 2024-07-11

**Authors:** Samantha Sutton, Alida Palmisano, Erika Ginsburg

**Affiliations:** 1 Samantha Sutton Ph.D. Leadership Coaching, Boston, MA 02468 USA; 2 General Dynamics Information Technology (GDIT), Falls Church, VA 22042 USA; 3 Center for Cancer Training, National Cancer Institute, National Institutes of Health, Rockville, MD 20850 USA

**Keywords:** Postdoctoral trainees, Career coaching, Science career, Biomedical research, Mentorship, Professional skills

## Abstract

Despite making strides in gender equality, women in Science, Technology, Engineering, Mathematics, and Medicine (STEMM) continue to face a persistent underrepresentation in leadership roles. In an effort to reverse this trend, the National Cancer Institute created the Sallie Rosen Kaplan (SRK) Postdoctoral Fellowship, a year-long coaching-based leadership training program. The SRK program aims to empower women to explore careers across a broad range of fields, including academia, industry, and government, and to excel in leadership positions in those fields. Analyzing a decade of data from 111 participants, we describe the positive impact of the SRK program on participant's self-reported capabilities. Increased self-confidence, improved time management and work/life balance, enhanced goal-setting and attainment skills, and strengthened communication and relationship-building abilities are highlighted as statistically significant outcomes. Moreover, the program’s emphasis on coaching, mentorship, peer cohort support, and building lasting professional relationships also contributed to high ratings for satisfaction and value of the program. Successful programs like SRK can serve as a model for institutions striving to close gender gaps in leadership.

**Supplementary Information:**

The online version contains supplementary material available at 10.1007/s13187-024-02466-1.

## Introduction

Despite decades of effort, there are still disproportionally fewer individuals who self-identify as women (referred to as “women” for the sake of brevity in this paper) in leadership roles in Science, Technology, Engineering, Mathematics, and Medicine (STEMM) disciplines in general [1, 2] and in biomedical research in particular [3]. For example, recent population studies have shown that women earn a higher percentage of Bachelor and Doctorate degrees in the biological sciences than men but fall below parity at the postdoctoral level [2]. Furthermore, other studies have shown that underrepresented minorities exit the academic track at the postdoctoral stage in disproportional numbers [4]. The representation of women falls even further for each step up the academic leadership ladder, as researchers move to Assistant, Associate, and, finally, Full Professor [2].

Similar trends occur in industry roles, with women holding only 8.2% and 10.4% of CEO roles in S&P 500 companies [5] and Fortune 500 companies [6], respectively. Women are underrepresented in STEMM leadership roles not because they are innately less suited to these fields [7, 8]. Research shows that women choose to leave STEMM career paths because of challenges originating from societal and systemic structures that impact individual perceptions and choices [2, 8–12]. Among the many published efforts to help address those challenges, there are two main programmatic approaches. The first is to provide opportunities for women to build leadership skills that could help them to be more successful, such as in communication, goal setting, management, relationship-building, and leadership style, among others [13–20]. A second approach involves instigating systemic change that mitigates or eliminates the institutional challenges faced by women. This can include addressing bias in hiring and recruitment, reevaluating how funding and promotion decisions are made, changing institutional culture, adding policies that support family commitments, and reducing bias in teaching and service requirements, among other approaches [2, 8].

The Sallie Rosen Kaplan (SRK) Postdoctoral Fellowship for Women Scientists in Cancer Research at the National Cancer Institute (NCI; an Institute of the National Institutes of Health of the U.S. Federal Government) was established in 2000 to support women in advancing through the academic career track. It was funded through the estate of Sallie Rosen Kaplan, who had a deep interest in the education of women. The initial version of the fellowship provided a one-time stipend inducement to bring women biomedical postdoctoral researchers to the NCI. However, after the first decade of the program, it became clear that there were opportunities to improve the program in order to have a greater impact. First, it was observed that simply recruiting women postdoctoral fellows to the NCI was not addressing broader challenges that those women faced in advancing along biomedical career paths. It has been shown that women with STEMM PhDs who have participated in leadership and management training are more likely to obtain mid/upper-level leadership roles [21]; however, studies have also found that postdoctoral fellows receive little of such professional training [22]. Therefore, in 2013, the SRK program was revamped to become a year-long program offering training in key leadership skillsets. Additionally, given the prevalence of STEMM professionals interested in careers outside of academia [23], it became clear that the program should also focus on supporting the advancement of women in a diverse range of STEMM pursuits. Thus, the scope of the program was expanded in 2013 to support fellows in being successful at high levels of leadership within academia, industry, government agencies, and other biomedical fields.

We built the SRK program to focus on developing the following key leadership competencies in our fellows:

- **Self-confidence.** Prior research has shown that girls and women have lower confidence in their abilities in science and math than boys and men [24, 25], and in their abilities to succeed in a STEM-related profession [26, 27], even when compared to similarly-ranking men [28]. Prior research has also shown that women are underrepresented in fields where raw innate talent is believed to be required for success, such as in STEMM fields, because women are societally stereotyped as not possessing such innate talent; this stereotype-threat impacts self-confidence irrespective of objective measures of success [29]. This lack of self-confidence has been shown to be correlated with adverse gender-based career outcomes, including a pay gap (despite no difference in performance) and advancement in STEMM fields [26, 28]. Thus, the central goal of our program was to build fellow self-confidence in their STEMM-related talents.

- **The ability to manage time and create work/life balance.** It has been shown that one of the main reasons women doctoral students shift away from pursuing academic leadership positions is the perceived challenges of reconciling the time demands of an academic job with those of other life priorities, most notably those of raising children [1, 10]. However, we found that most postdoctoral fellows have received minimal training in time management techniques in their careers and hypothesized that growing this skillset could enable them to create space for multiple life priorities, even in demanding environments. Thus, one of the goals of our program was to strengthen key time management skills such as prioritization, self-agency, planning, and delegation.

- **Individual goal setting.** Goal setting theory was first formulated in 1990, positing that goal setting leads to better performance than not setting goals [30]. Subsequently, goal setting has been shown to increase motivation, learning, self-efficacy [31], and network-building [32, 33]. Goal orientation has also been shown to correlate with career success [33]. As a result, we incorporated short- and long-term goal-setting and attainment into the SRK curriculum.

- **Strong relational and communication skills.** The success of STEMM leaders comes in part from their ability to interact productively with a variety of other people in their workplace, including peers, managers, subordinates, advisees, mentees, organizational staff, and outside agents [34, 35]. Thus, we focused on relational skills such as having difficult conversations, emotional intelligence, and communication skills such as presentation and writing grant proposals.

- **Building a supportive professional network.** Professional networks, both peer and mentor-based, are important for learning and knowledge transfer, exposure to opportunities, and advocacy for advancement [36–38]. In particular, mentoring has been shown to promote the persistence and progression of underrepresented minorities in STEMM fields [39]. Postdoctoral fellows can struggle to build such professional networks, as they typically enter their institutes asynchronously and not as a part of a cohort, leading to highly variable exposure to peer groups; additionally, because of the apprenticeship model of the postdoctoral position, they often have no additional mentor apart from their principal investigator [19]. Thus, we provided both peer and mentor networks to fellows during the SRK program.

To best promote the development of all the skillsets listed above, we chose to center the program on executive/leadership coaching. As one of the primary methods for developing leaders in industrial settings [40], coaching has been shown to improve performance and goal setting [41] and assist in the development of new outlooks and behaviors [42]. Supporting the leadership coaching curriculum, we included additional topical workshops, mentorship opportunities, grant-writing and presentation exercises, and the formation of a strong and supportive fellow cohort. We collected and analyzed ten years of participant data to determine the impact of the program.

## Methods

### Program Participants

Participants were postdoctoral research fellows who self-identified as women, performing cancer research at the NCI. Participants had received their highest degree (typically PhD) between 1 and 7 years prior to applying to the SRK program. Participants performed research at one of the three NCI Maryland campuses: Bethesda, Shady Grove, or Frederick. Participants were either basic science researchers, epigeneticists, or population scientists and were competitively selected from a self-nominated application package. The package contained a personal statement of professional goals, including non-scientific career aspirations and expectations for the program, a professional history and a resume highlighting professional accomplishments, a statement of work environment describing any limitations currently faced, and a note of any special circumstances. In addition, applicants were required to submit recommendation letters from their primary research mentor and laboratory/branch director, which indicated support for the required activities, benefits to the applicant, and acknowledgement of the time commitment of the program. Applications were submitted by the applicant through an online system and scored by a review committee on the content and quality of the materials submitted. Initially, ten applicants were selected to participate in the program for cohorts 1–4. The program proved to be popular, and, as a result, the cohort size was increased to 12 for subsequent years.

### Program Commitment

Upon acceptance, each fellow signed a commitment letter indicating they would participate fully in all scheduled workshops, activities, and assignments. This commitment emphasized the interconnected nature of all the components of the program and how each one builds upon the others to create the cumulative value of the program. Over the course of the year-long program, excused absences for personal and family reasons were allowed, but in order to graduate from the program, participants could accrue no more than two. Additionally, the NCI staff running the SRK program held themselves responsible for the success of the program by setting clear expectations, monitoring attendance and participation, being present for the sessions, and serving as unofficial mentors throughout the program.

### Program Curriculum

The program lasts 10 months (November through August) and aims to teach fellows a skillset that will help them be successful in future leadership roles in the biomedical sciences. This skillset includes building self-awareness and self-confidence, goal setting, developing strong relationships, conflict resolution, time management, cultivating accountability, situational leadership, and enhancing emotional intelligence, among other topics. The SRK program teaches these skillsets via five main program components: leadership coaching, mentorship, peer support, additional skill building, and grant writing (Table 1).

**Table 1:**
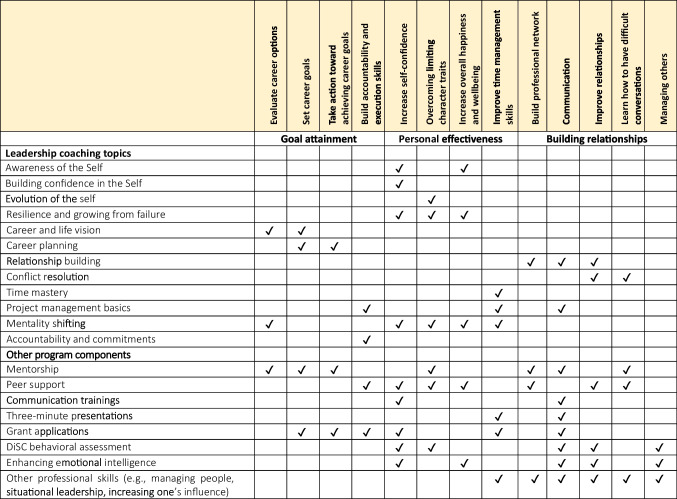
The components of the SRK program and the skills that fellows learn in each

### Leadership Coaching

The core of the program is a 9-month coaching curriculum designed and coached by Samantha Sutton, PhD and based on the co-active coaching model. Co-active coaches teach tools and approaches for personal growth and then empower participants to leverage their own innate strengths and resources to find solutions. The SRK coaching curriculum consists of two modules. Module 1 builds a strong internal leadership core of self-awareness, self-confidence, and a clear and compelling articulation of career goals. Module 2 leverages this core to teach fellows how to positively shape their external environment, including time management and building professional and personal relationships, to support their career ambitions and allow them to successfully work in whatever environment they choose. Coaching topics are further detailed in Table 2.

**Table 2:**
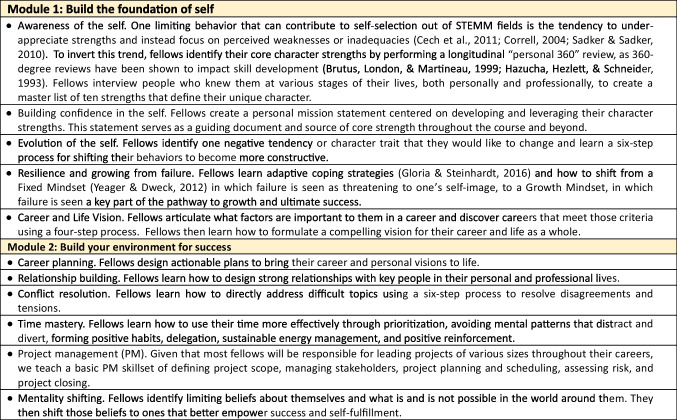
Topics covered in the coaching component of the program

Each module begins with an 8-h in-person interactive workshop that consists of instruction, coaching, reflection, and group work. Workshops are held in January (Module 1) and April/May (Module 2) and are each followed by seven 2-h sessions conducted over videoconference. All workshops and sessions include a required homework assignment, due 3 days prior, in response to which fellows receive individual feedback.

Throughout both modules, fellows design weekly commitments around tasks they will accomplish, both personally and professionally, to help achieve desired outcomes in their lives. Fellows receive coaching and feedback on their performance from an accountability coach, Tanara Martin, MEd, LCPC, with the intention of building accountability and driving performance.

### Mentorship

In order to gain expert career advice in their chosen field of study and network with potential role models, fellows select a mentor from a pool of accomplished women with scientific careers in academia, industry, and government. Mentor engagement varied by fellow and typically included monthly meetings (either in-person or via phone or videoconferencing) and group events consisting of networking opportunities, informal social gatherings, and presentations. Key contributions provided by mentors include sharing success strategies, celebrating fellow accomplishments, and acting as a source of support.

### Peer Support

As an additional learning aid and source of reflection and support, fellows were assigned into peer groups of 3–4 fellows (“buddy groups”). These groups met at varying frequencies, either in-person or via phone or videoconferencing, and shared assignments and commitment results with each other. Peer group members were charged with holding each other accountable for doing their best work during the program, even when working through difficult topics.

### Communication and Presentation Skills

Fellows also participated in two workshops on communication and presentation skills to improve the effective delivery of their scientific message through body language and tone. The lessons were put into action during a Three-Minute Talk, modeled after the Three-Minute Thesis,^1^ where fellows described an aspect of their research in a single slide within 3 min. Research and program mentors were invited to provide constructive feedback on the presentations.

### Writing a Grant Proposal

The fellows are invited to apply for a competitive grant to purchase materials for a novel research project intended to generate preliminary data for a future grant application. The application format follows the NIH K99/R00 Pathway to Independence Award, and reviewers are asked to score applications following the NIH scoring system, which uses a 9-point scale for overall impact and evaluates criteria including career development and research plan (significance, innovation, and approach). The opportunity is open to current SRK fellows as well as all SRK alumni who are still at the NCI. All fellows who apply receive a critique of their application. This grant proposal writing exercise is valuable even for fellows who do not aspire to go into academia, as fellows learn how to conceptualize a new idea and to write clearly and convincingly.

### Additional Skill Building

To offer a core set of tactical skills, fellows attended additional customized 2-h seminars led by the NCI Office of Workforce Planning and Development (recently renamed to the Office of Workforce Strategy and Effectiveness). Seminar topics included the DiSC behavioral/work style assessment, enhancing emotional intelligence, managing people, situational leadership, managing change and transition, and increasing one’s influence. These seminars featured instruction as well as individual work and reflection.

### Survey Instrument

In order to measure the success of the SRK program, we developed and administered pre-program and post-program surveys. The original version of the survey was piloted on cohorts 1 and 2 (data not shown). The fellows indicated the SRK program helped them improve self-confidence. Based upon these initial responses, we designed a more comprehensive survey to better understand the program impact. Questions for the revised survey were developed based on the stated goals of the program. The survey included questions on program expectation, self-perception of skillsets, confidence in chosen career paths, and strength of interpersonal relationships. The survey also included open-ended questions intended to gauge program satisfaction.

The survey was distributed online using SurveyMonkey to fellows accepted to the program. Baseline data were collected within 1 week of acceptance into the program (pre-program survey; Supplementary Table 1). The survey was administered again within 1 week of completion of the program (post-program survey; Supplementary Table 1) in order to measure longitudinal growth over the course of the program. Invitations to respond to the survey were distributed by email and responses were anonymous and voluntary. Respondents had 1 week to complete the survey, and one reminder was sent prior to the due date.

### Data Analysis

We summarized the survey responses through descriptive statistics and analyzed the pre-program and post-program responses for each question. Using R *stats* and *ggsignif* packages, we performed a Wilcoxon test and identified which differences were statistically significant (*p *values of <0.05 were considered statistically significant). For responses with a defined set of discrete values, the R package *ggplot* was used to produce a combination of boxplots and violin plots displaying the distributions of pre-program and post-program responses in cohorts 3 to 10. A violin plot depicts distributions of the data with (mirrored) probability density curves. Each curve corresponds to the smoothened frequency of data points in that region, wider sections represent a higher probability of a given value. In combination with boxplots (which show summary statistics such as median and interquartile ranges), the violin plots reveal valuable information about the full data distribution shape and skewness. For open-ended questions with narrative responses, in order to show recurrent topics and keywords, we extracted the qualitative themes, and using the R package *wordcloud,* created a summary graphic where the size and color of the words corresponded to their frequency of occurrence.

### Post-program Connections

After each cohort completes the program, we maintain professional connections with the alumni as they move through their careers. At the start of the program, fellows are encouraged to join the SRK LinkedIn group. This private group allows fellows to network with program alumni and to keep up to date on each other’s professional trajectories.

Furthermore, we hold an annual SRK Career Day, which brings alumni back to the NCI to meet with current fellows. A keynote speaker is invited to present on a topic of interest to the fellows; examples of topics include the role of mentorship, combating imposter syndrome, life after postdoc, and unconscious bias. Alumni also participate in career panels which are designed to expose fellows to career options in academia, government, industry, and other biomedical fields. The career panels are an opportunity for fellows to hear what the panelists learned from the SRK program and how it helped them progress to the current stage of their professional trajectory.

## Results

Of the 111 fellows who participated in the SRK program, all officially completed the program. While a few fellows left the NCI to take another position before the official end of the program, they attended any remaining sessions as time permitted.

We surveyed fellows at the beginning (“pre-program survey”) and end (“post-program survey”) of the program about their skill growth experiences and about their scientific career interests and challenges. The surveys were voluntary, and 95.6% (86 respondents) and 92.3% (84 respondents) of fellows submitted answers to the pre-program and post-program surveys, respectively. In the following sections, we present key responses pertaining to self-perception of skills and capabilities, careers in science, and overall impressions of the program. All the collected answers to each individual question can be found in the supplementary material 2.

### Self-confidence

**Fig. 1:**
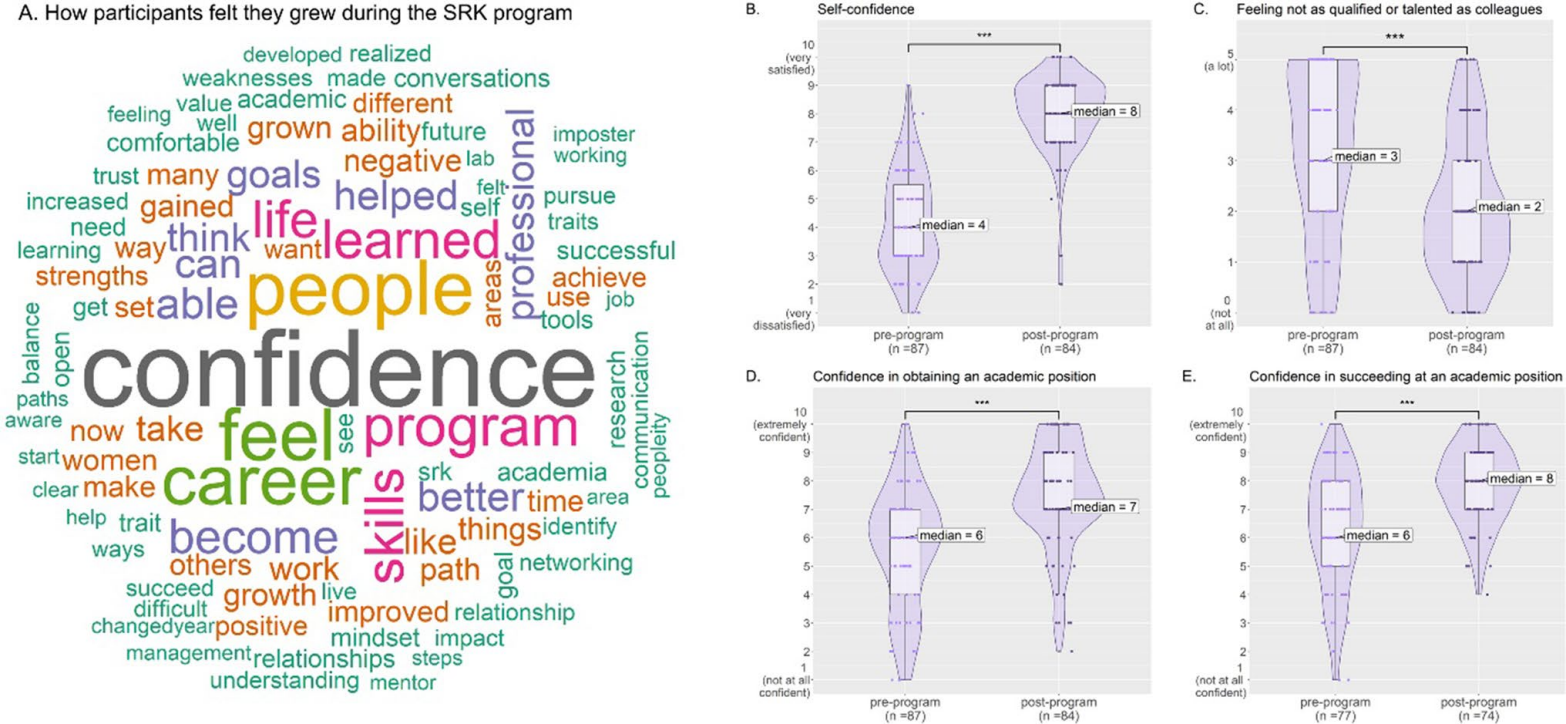
**A** Word cloud created from the open-ended responses of fellows in cohorts 3–10 to the post-program survey question “In what ways do you feel you have changed or grown as a result of your participation in the program?” The size and color of the words correspond to their frequency. **B–E** Responses to survey questions evaluating fellow self-confidence, including self-perception of self-confidence (**B**), feeling not as qualified or talented as colleagues (**C**), confidence in obtaining an academic position (**D**), and succeeding in an academic position (**E**). Each panel shows the individual responses (dots), violin plots (darker purple, displaying the smoothened frequency of data points in that region of the *y*-axis), and box plots (lighter purple, displaying the median and interquartile range) comparing the pre-program and post-program ratings. Strongly significant changes in post-program outcomes are marked with *** in the plot (*p* ≤ 0.001)

One of the central goals of the SRK program was to help fellows become more confident in themselves and their abilities, as that has been shown to lead to better career outcomes (see "Introduction"). The concept of self-confidence showed up in several of the survey results. When asked the open-ended post-program survey question “In what ways do you feel you have changed or grown as a result of your participation in the program?”, the single word most used was “confidence,” mentioned by 53.5% of the 84 respondents (Fig. 1A).

Fellows were asked to rate on a scale of 1 (very dissatisfied) to 10 (very satisfied) their satisfaction with their abilities in several areas, including self-confidence. The median rating of “Self-confidence” increased from 4 in the pre-program survey to 8 in the post-program survey (statistically significant, *p *value ≤ 0.001, Fig. 1B).

Additionally, fellows were asked to rate on a scale from 0 (not at all) to 5 (a lot) how strongly different considerations negatively impacted their perceptions about their abilities to succeed in an academic professorship. The median of the pre-program and post-program answers to the question “Feeling not as qualified or talented as colleagues” decreased from 3 to 2 (*p *value ≤ 0.001, Fig. 1C), indicating a statistically significant increase of confidence when self-comparing to peers.

Finally, when asked to rate on a scale of 1 (not at all confident) to 10 (extremely confident) “How confident are you that you could obtain an academic position in the future?” and “How confident are you that you could succeed in an academic position in the future, once you have obtained it?”, the median response increased from 6 for both questions (pre-program) to 7 and 8 (post-program), respectively (*p *values ≤ 0.001, Fig. 1D, E), indicating that the fellows became more confident in their competitiveness as academic researchers over the course of the program.

### Time Management, Work/Life Balance, and Goal Setting

One of the reasons women shift away from pursuing advanced careers in science is the challenge of reconciling the time demands of a scientific job with other life priorities (see "Introduction"). A goal of the SRK program was to strengthen fellow time management skills and strategies so that they could find a fulfilling balance of their work and personal lives.

**Fig. 2:**
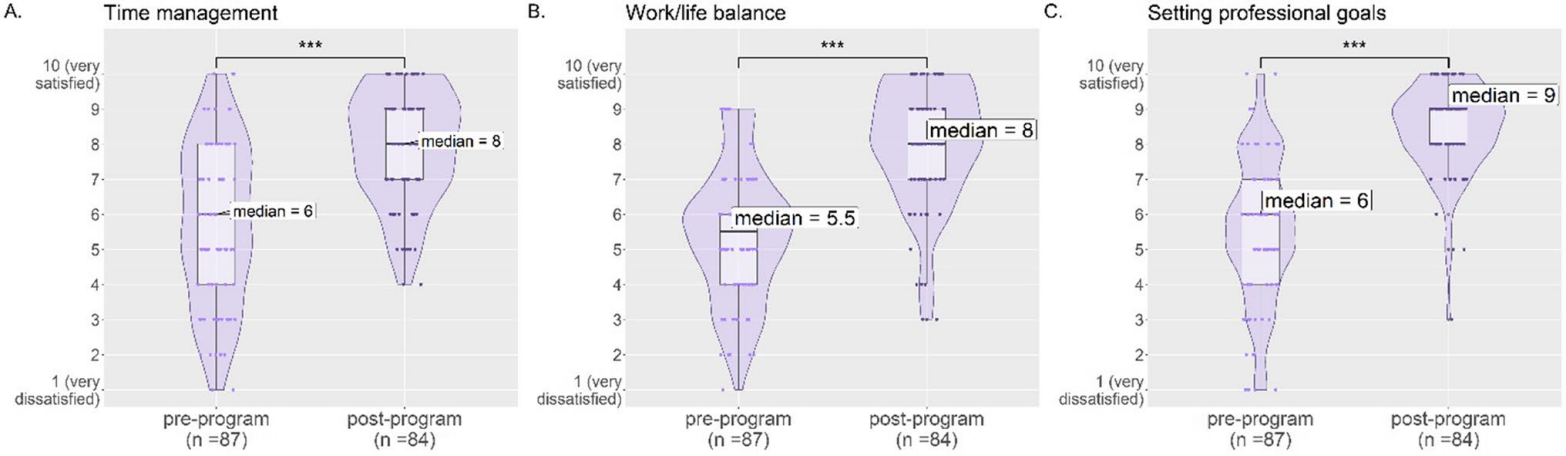
Responses to survey questions assessing time management (**A**), work/life balance (**B**), and setting professional goals (**C**). Each panel shows the individual responses (dots), violin plots (darker purple, displaying the smoothened frequency of data points in that region of the *y*-axis), and box plots (lighter purple, displaying the median and interquartile range) comparing the pre-program and post-program ratings. Strongly significant increases in post-program outcomes are marked with *** (*p* ≤ 0.001)

Fellows were asked to rate on a scale of 1 (very dissatisfied) to 10 (very satisfied) their satisfaction with their abilities in multiple areas including time management and work/life balance. The median rating of “Time management” increased from 6 in the pre-program survey to 8 in the post-program survey (*p *value ≤ 0.001, Fig. 2A), and the median rating of “Work/Life balance” increased from 5.5 to 8 (*p *value ≤ 0.001, Fig. 2B).

Given that goal setting has been linked with positive outcomes such as motivation, self-efficacy, and career success (see "Introduction"), fellows were also asked to rate their satisfaction in setting professional goals on a scale of 1 (very dissatisfied) to 10 (very satisfied). The median rating of “Setting professional goals” increased from 6 in the pre-program survey to 9 in the post-program survey (*p *value ≤ 0.001, Fig. 2C). Additionally, fellows indicated in the post-program survey that the program helped them “Set goals for my career” (81% of respondents), “Evaluate potential career paths” (79% of respondents), and “Take action toward achieving career goals” (88% of respondents) (Supplementary Fig. 1).

### Relational and Communication Skills

**Fig. 3:**
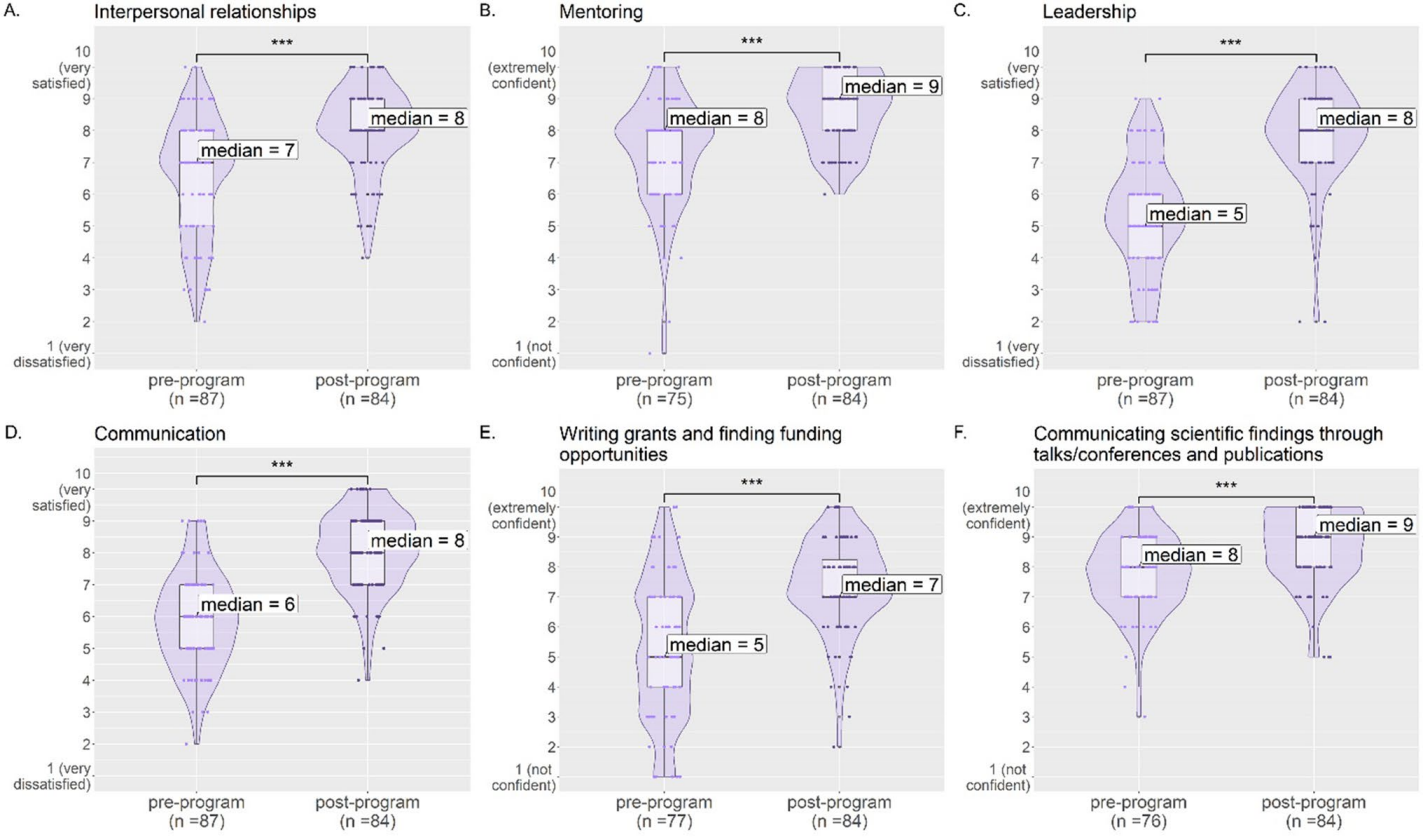
Responses to the survey questions evaluating fellow satisfaction and confidence with their skills in interpersonal relationships (**A**), mentoring (**B**), leadership (**C**), communication (**D**), writing grants and finding funding opportunities (**E**), and communicating scientific findings through talks/conferences and publications (**F**). Each panel shows the individual responses (dots), violin plots (darker purple, displaying the smoothened frequency of data points in that region of the *y*-axis), and box plots (lighter purple, displaying the median and interquartile range) comparing the pre-program and post-program ratings. Strongly significant increases in post-program outcomes are marked with *** (*p*  ≤ 0.001)

Given the importance of strong professional relationships in career advancement (see "Introduction"), the SRK program introduced methods to help fellows build relationships. Fellows were asked to rate on a scale of 1 (very dissatisfied) to 10 (very satisfied) their perception of their abilities in multiple areas. The median rating of ability in “Interpersonal relationships” increased from 7 in the pre-program survey to 8 in the post-program survey, “Mentoring” increased from 8 to 9, and “Leadership” increased from 5 to 8 (*p *values ≤ 0.001, Fig. 3A–C). In addition, fellows indicated in the post-program survey that the program helped “Improve my interpersonal relationships” (67% of respondents) and “Build my professional network” (82% of respondents) (Supplementary Fig. 1).

A key element of building relationships is the ability to communicate. When asked to rate on a scale of 1 (very dissatisfied) to 10 (very satisfied) their “Communication” abilities, the participant median rating increased from 6 in the pre-program survey to 8 in the post-program survey (*p *value ≤ 0.001, Fig. 3D). Fellows were also asked how confident they felt in competencies that involve a strong communication component such as “Writing grants and finding funding opportunities” and “Communicating scientific findings through talks/conferences and publications.” Median ratings increased for all of these actions between the pre-program and post-program surveys (with significant *p *values ≤ 0.001), increasing from 5 to 7 for writing grants and 8 to 9 for communicating through conferences and publications (Fig. 3E, F). Additionally, fellows reported in the post-program survey that the program helped them “Learn how to have difficult conversations” (57% of respondents) (Supplementary Fig. 1).

### Career Paths in Biomedical Science

In order to understand their career goals, fellows were asked to rank the top eight career paths they were interested in pursuing. In pre-program surveys, more than 75% selected one of their eight top choices to be “Research in biotech/pharma/other industry setting,” “PI in academia (research-intensive),” or “PI in academia (teaching and research).” The next highest selected careers of interest were “Other research in academia,” “Bench science in government,” and “Science consulting,” with more than 50% of fellows ranking them in the top eight (Supplementary Fig. 3).

**Fig. 4:**
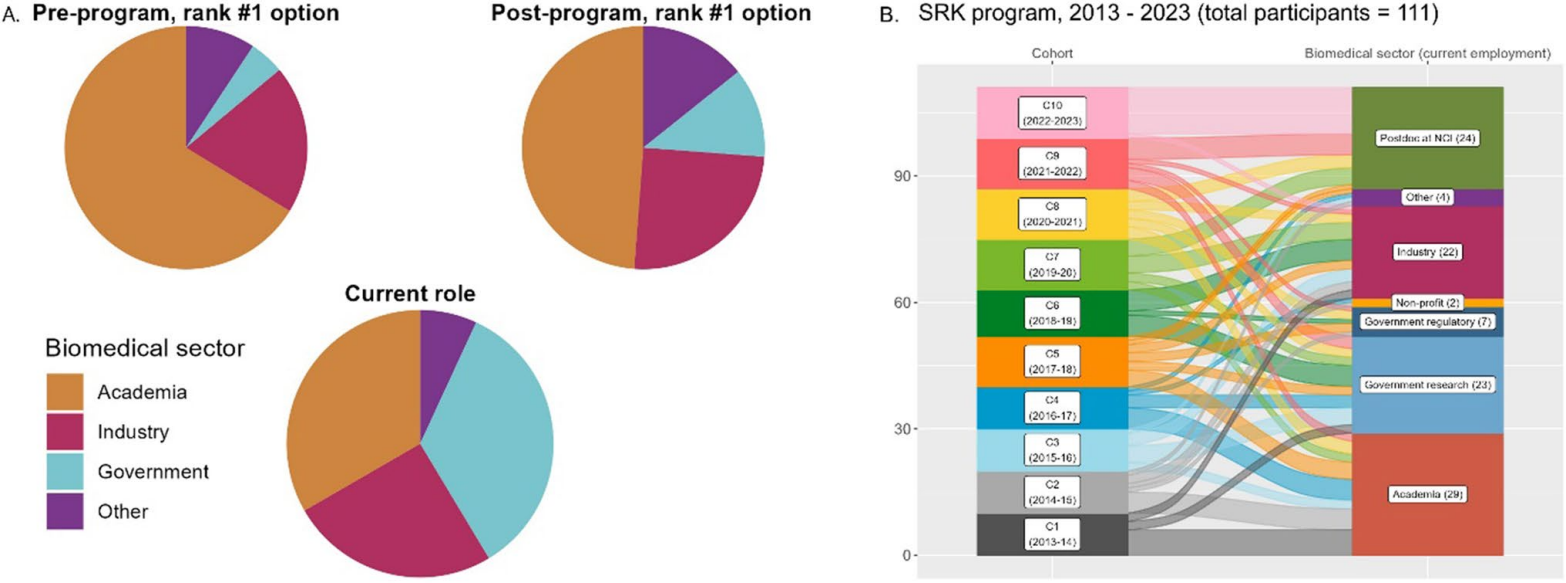
Participant career preferences and choices. **A** The highest-ranked career paths selected by SRK fellows in pre- and post-program survey results (top row) and the actual sectors in which fellows were employed as of December 2023 (bottom row). **B** Alluvial plot showing the 111 fellows from cohorts 1–10 (left column) matched to the sector in which they were employed as of December 2023 (right column)

Figure 4A shows the first choice career path of SRK fellows. In pre-program surveys, 66% of respondents chose an academic career path as their first choice, with 20% choosing industry paths, 9% choosing government paths, and 5% choosing other paths. In post-program surveys, 49% chose academic paths, 25% chose industry paths, 14% choosing government paths, and 12% choosing other paths. These results indicate a decrease in interest in academia and an increase in other career paths.

Moving beyond interest to attainment, we obtained and analyzed the actual roles of the 86 SRK fellows who had completed their NCI postdoctoral fellowships as of December 2023. Of those alumni, 34% were working in government, 33% were working in academia, 25% were working in industry, and 7% were working in other fields. The current roles of all 111 SRK cohort fellows can be seen in greater detail in Fig. 4B. All (100%) of the SRK program fellows are currently employed in STEMM roles.

### Satisfaction with the Program

**Fig. 5:**
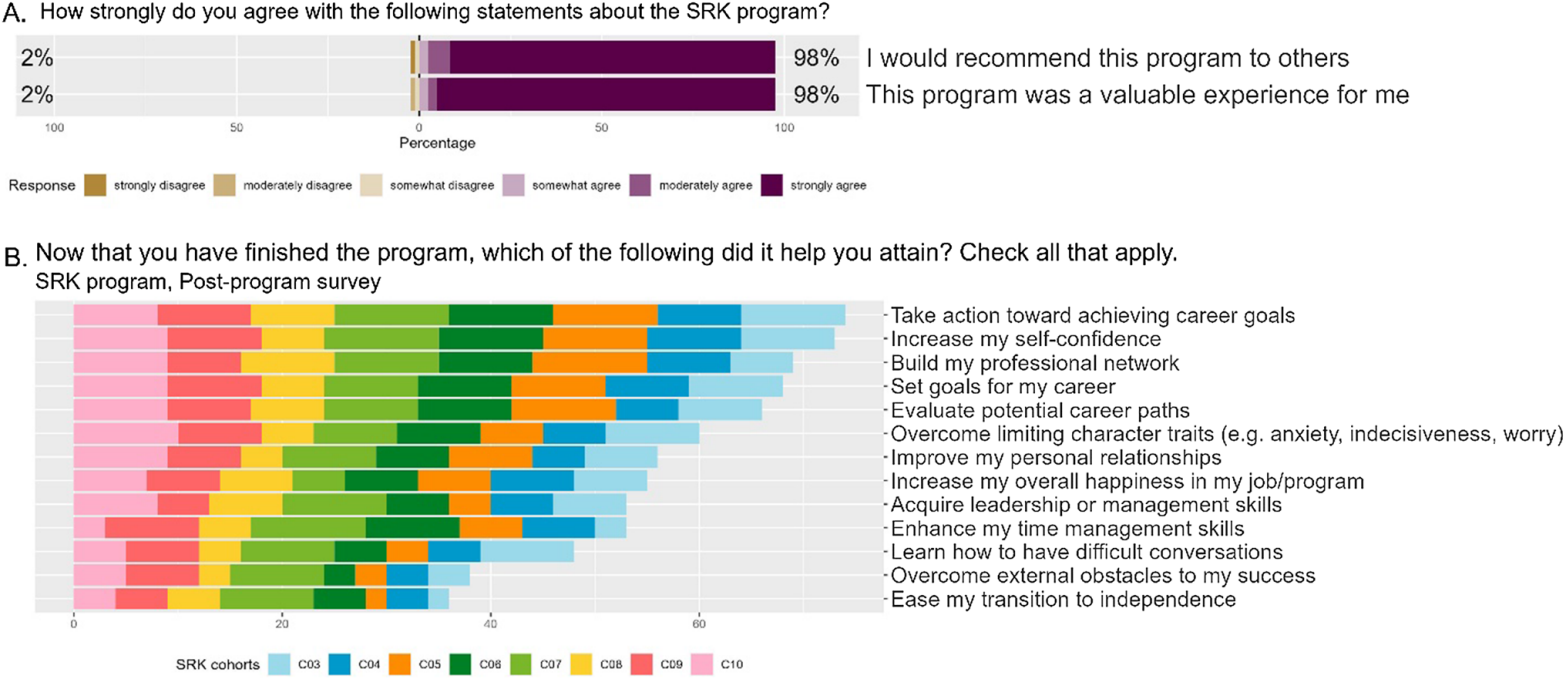
**A** Responses to post-program survey questions about fellow satisfaction with the SRK program. The bar chart shows the results using a six-point Likert scale. **B** Responses to the post-program survey question about competencies the SRK program helped fellows attain. Each row displays the number of fellows in each of the cohorts that selected that option

In the post-program survey, respondents were asked about their satisfaction with the program on a six-point scale from “strongly disagree” to “strongly agree.” Of the post-program survey respondents, 98% agreed that they would recommend the SRK program to others, with 89% of the respondents selecting “strongly agree” (Fig. 5A). Additionally, 98% agreed that the program was a valuable experience, with 93% of respondents selecting “strongly agree.” The few fellows who gave the lowest ratings highlighted mismatched expectations between the program curriculum and their experience of the program.

The responses to the post-program survey open-ended growth questions “In what ways do you feel you have changed or grown as a result of your participation in the program?” and “Which elements of the program were most valuable to you?” were overwhelmingly positive about the coach-facilitated content and the value of having a coach who helped them see themselves and their situation in new ways. Skills that resonated with most of the fellows were building confidence, improving personal relationships, feeling empowered to “dream” about their career goals, and learning strategies to overcome negative limiting beliefs. Communication workshops, oral presentations, and grant applications were also mentioned as elements of the program that provided value to SRK fellows.

Many fellows mentioned the positive impact of the professional connections the program facilitated both with accomplished senior scientists and fellow participants. Many SRK fellows highlighted the importance of the personal accountability they felt toward the other fellows in their cohort and the value of having a shared experience with other ambitious and skilled women scientists. Fellows also commented that they felt that connections made during SRK provided immediate support during the program and fostered long-term relationships that will continue throughout their scientific career progression.

To obtain a global snapshot of the breadth of impact of the course, fellows were presented with a list of 13 leadership competencies and asked to select which ones the SRK program helped them attain. More than 80% said that the program helped them take action toward achieving career goals, increasing self-confidence, building their professional network, and setting goals for their career. The next set of results, selected by more than 65% of fellows, included evaluating potential career paths, overcoming limiting beliefs, improving personal relationships, and increasing overall happiness in their jobs. All responses are shown in Fig. 5B.

## Discussion

One of the main goals of the SRK program is to support women both in staying in STEMM and in attaining high levels of leadership in their chosen STEMM fields. At the time of writing this paper, 100% of course participants have remained in STEMM roles across a variety of sectors.

While many factors have likely contributed to this outcome, fellow satisfaction with their SRK experience suggests that the SRK program could be a contributing factor. In analyzing the data from the past SRK programs (2015–2023), we found that fellows enter the program with the intention to grow their leadership skillset, increase their self-confidence, build their professional network, set and attain career goals, and improve their communication skills. After participating in the program for a year, the vast majority of fellows reported that they “agree” that they would recommend the program to others and “strongly agree” that the program was a valuable experience. Anecdotally, during the first coaching workshop, fellows often explain that they chose to participate because they heard from alumni that the program would “change their life” and that they were impressed by the changes they witnessed in SRK alumni. We have also informally discussed program outcomes with research mentors of SRK participants and learned that many of them have observed visible changes in the participants. For example, one noted that a participant from their lab “seemed to thrive in the support group structure provided by the SRK program and benefited from increased self-awareness and confidence after interacting with the life coach… [she] realizes that her ambitions are within her grasp, and she is capable to achieve them.”

The high course satisfaction is likely due to a measurable self-reported increase in several key capabilities. Fellow self-confidence significantly increased, as measured through several survey questions. As one anonymous respondent said, “I’ve gained more self-confidence as I’ve realized that I already have the skills to be a leader.” Much of the research mentor feedback highlighted a noticeable increase in self-confidence as well, with one saying, anonymously, “She is able to articulate her thoughts in a more confident manner, [and] assert herself more.” Given the published importance of self-confidence in retention in STEMM fields, it is our hope that this confidence will empower fellows to strive for roles that might have previously seemed unreachable either due to the competitive nature of the role, or the demands of the role.

Participant time management skills and corresponding ability to strike a satisfactory work/life balance also increased. According to one survey recipient, “I stopped feeling bad about striving [for] a good work/life balance. I learned to celebrate even the smallest things I achieve. I can use my time more effectively.” Many research mentors noted the same trend, commenting that their participants had learned “how to balance and prioritize workloads” and were “demonstrating more confidence and better work/life balance.” Given that work/life balance is one of the main cited reasons that women leave STEMM fields, it is our intention that this improved skillset will enable fellows to have both satisfying professional and personal lives and not to have to choose between the two.

Fellows also reported that the course helped them in goal setting, specifically in setting goals for their career, evaluating potential career paths, and taking action toward achieving goals. As one survey respondent said, “I am more confident in my abilities, and am better at setting attainable goals. I am also more likely to take risks and push myself in terms of my career.” Several research mentors noted likewise, with one commenting that “She became more proactive about submitting abstracts/applications for conferences and grants. She became more proactive about her research plans and continuing education.” Given the importance of goal-setting in motivation, learning, and career outcomes, it is our hope that fellows can leverage these goal-setting skills to aim for (and attain) more ambitious leadership roles within STEMM fields.

The main goal of the course was to improve communication and relationship-building skills. Fellows reported that the course helped them in communication, building interpersonal relationships, mentorship, and network-building; having difficult conversations; and writing grants and finding funding opportunities. One survey respondent noted that “My communication skills improved, I learned how to have an efficient and compelling conversation according to my expectation and needs, and I became braver in speaking out for myself in a crucial conversation. [Now] I know how to improve a relationship that I care about.” Multiple research mentors commented on the participant's improved communication skills, with one saying “I think she recognizes her strengths better. She is more likely to participate in scientific discussions.”

A key design feature of the SRK program is its coaching core. The program is structured such that the SRK coach progressively builds a deep understanding of each of the fellows and through this is able to help each fellow grow in a way uniquely suited to their own needs and situations. A key part of this growth is helping fellows to see the challenges they face from a new, more empowering perspective, which allows them to identify more effective approaches to solve those challenges.

In addition to the skillsets attained during the course, one of the highly cited benefits of the course is the relationships formed both with cohort fellows and mentors. For example, one fellow said, “I gained a community of female scientists at the same stage of their career - those relationships will likely serve me long into the future.” During the SRK course, fellows experienced significant life events such as pregnancies, births, marriages, divorces, the loss of loved ones, or the loss of a job. The buddy groups and cohorts were often mentioned as a primary source of support for fellows going through such life events. In addition to the relationships formed with peers, many SRK fellows cited the perspective and support they were able to obtain from their SRK mentor. It is our intention that providing strong, and hopefully lasting, relationships with other women in STEMM fields will provide our fellows with the support, learning, knowledge-transfer, and advocacy that has been shown to be instrumental in successful STEMM careers.

From the survey results, we observed a decrease in interest in academic professorships among fellows during their SRK year. This trend has been previously observed in PhD role outcome studies [47]. While there are likely many forces at play in this trend, the SRK program likely showcased a wider variety of STEMM career paths to fellows and challenged them to deeply introspect about which would be most fulfilling to them.

There are two main limitations of our survey methodology. The data measuring the success of the program is collected via self-assessment, which may or may not reflect the skillsets that fellows can deploy in their current and future jobs. Additionally, we measure course impact directly after the course ends. We suspect that the skills learned during the course will persist into the future, but we do not measure this directly. Anecdotally, we have heard from course alumni that they not only use the SRK skillsets in their own careers but teach them to their mentees as well.

While we have spent the past 10 years refining the course itself, future improvements to the course could include greater involvement of alumni in the form of follow-on topical coaching offerings, additional networking opportunities, and participation by alumni in course sessions to share real-world experiences and lessons learned. Other areas for improvement could include additional interactions among the alumni and the mentors; this would increase not only camaraderie among the individuals but also help them expand their networks.

In conclusion, despite progress over decades, women still trail behind in leadership roles in STEMM both within academia and industry. The SRK fellowship strives to lessen this gap by building key leadership competencies in women during an early time in their scientific careers when key career decisions are made: the postdoctoral fellowship. These competencies include self-confidence, time management, goal setting, communication, and creating a supportive professional network. Eight years of participant data show positive outcomes in all of these competencies. It is our hope that the success of the SRK program can serve as a model to other institutions that are looking to equip their women scientists with the skills that can help close the leadership role gap, thereby fostering innovation and propelling scientific advancement via a diverse and inclusive workplace.

## Electronic Supplementary Material

Below is the link to the electronic supplementary material.

Supplementary Material 1

Supplementary Material 2 (XLSX 59.2 KB)

## Footnotes

1 Footnote 1: For details, see the official 3MT website https://threeminutethesis.uq.edu.au
