# Supplementary material for: A Coaching-Based Leadership Program for Women Postdoctoral Fellows at the National Cancer Institute that Cultivates Self-confidence and Persistence in STEMM
Source: J Cancer Educ. 2024 Jul 11;39(5):507–19. doi: 10.1007/s13187-024-02466-1 (PMC11461782; doi:10.1007/s13187-024-02466-1)
Supplement: Supplementary file 1 — Supplementary Material 1 [file 13187_2024_2466_MOESM1_ESM.pdf]

Supplementary material of

A coaching-based leadership program for women post-doctoral fellows at the National Cancer Institute that cultivates self-confidence and persistence in STEMM

Samantha Sutton [+,1], Alida Palmisano[+,2], Erika Ginsburg [*,3]

1 Samantha Sutton Ph.D. Life Engineering, Boston, MA 02468, USA
2 General Dynamics Information Technology (GDIT), Falls Church, VA 22042, USA
3 Center for Cancer Training, National Cancer Institute, National Institutes of Health, Rockville, MD, 20850, USA

+ The authors wish it to be known tha Sutton and Palmisano should be regarded as Joint Co-First Authors.
*Address correspondence to: Erika Ginsburg (email: **ginsbure@mail.nih.gov**)

The present document contains supplementary material for the paper.
The document contains graphical representations of the answers given by SRK fellows to the pre-program and post-program surveys.
The raw data of the individual participants response to the survey's multiple-choice questions is provided via the publisher journal website as a separate supplementary spreadsheet.

Supplementary Table 1: The survey instrument.

Sallie Rosen Kaplan (SRK) Postdoctoral Fellowship for Women Scientists

Pre-program and post-program survey questions

Participants were asked to complete these surveys to help gain knowledge to improve future program development.
No identifiers were collected as part of the survey. The results are reported in aggregate form only.
Participation in the survey was voluntary and time-completion burden was estimated to be less than 10 minutes.

	Question	Response type	Responses / choices
Pre-program survey	What results are you hoping to attain from the Program? Check all that apply.	Multiple choices	Evaluate potential career paths<br>Build my professional network<br>Acquire leadership or management skills<br>Enhance my time management skills<br>Learn how to have difficult conversations<br>Improve my personal relationships<br>Increase my overall happiness in my job/program<br>Overcome external obstacles to my success<br>Overcome limiting character traits (e.g. anxiety, indecisiveness, worry)<br>Increase my self-confidence<br>Set goals for my career<br>Take action toward achieving career goals<br>Ease my transition to independence
Post-program survey	Now that you have finished the program, which of the following did it help you attain?		
		Open end, free text	Other (please specify)
Common between pre-program and post-program surveys	What career options are you currently interested in? There are 15 options. Please rank up to 8 options, with #1 being the option you are most interested in.	Single ranking choice, from 1 (most interested) to 8 (least interested)	PI in academia (research-intensive)<br>PI in academia (teaching & research)<br>Other research in academia<br>Research in biotech/pharma/other industry setting<br>Bench science in government<br>Teaching-intensive in academia (post-secondary)<br>Science education for the public<br>Science education for schools<br>Healthcare<br>Science writing or publishing<br>Science or education policy<br>Drug approval and production<br>Science consulting<br>Other (science-related)<br>Other (not science-related)
Common between pre-program and post-program surveys	Knowing that no relationship is perfect, how much improvement would be needed for you to have an ideal relationship with your PI? The further to the right you select, the more improvement is needed.	Numerical scale, from 0 to 100	
Common between pre-program and post-program surveys	How strongly do each of the following considerations negatively impact your perceptions about academic professorships?<br>Please rate these on a scale of 0 (not at all) to 5 (a lot).	Single ranking choice, from 0 (not at all) to 5 (a lot)	Negative experience as a Ph.D. student<br>Negative relationship with PI<br>Working in a collaborative environment<br>Feeling not as qualified or talented as colleagues<br>Bad job market<br>Poor funding environment<br>Career advancement issues<br>Monetary compensation<br>Concerns about excessive work hours<br>Interest in other careers<br>Geographic location issues<br>Family considerations (spouse/partner/children)
Common between pre-program and post-program surveys	Please list any additional considerations that were not included on the previous list.	Open end, free text	
Common between pre-program and post-program surveys	Please rate your satisfaction with your abilities in the following areas on a scale of 1 (very dissatisfied) to 10 (very satisfied).	Single ranking choice, from 1 (very dissatisfied) to 10 (very satisfied)	Time management<br>Leadership<br>Interpersonal relationships<br>Communication<br>Potential for growth<br>Work/life balance<br>Setting professional goals<br>Self-confidence
Common between pre-program and post-program surveys	On a scale of 1 (not at all confident) to 10 (extremely confident), how confident are you that you could:	Single ranking choice, from 1 (not at all confident) to 10 (extremely confident)	Obtain an academic position in the future?<br>Succeed in an academic position in the future, once you have obtained it?
	Explain your response to the previous question.	Open end, free text	
Common between pre-program and post-program surveys	Once you have obtained an academic position (or hypothetically if that is not your goal), how confident are you that you would be able to succeed in each of these areas:	Single ranking choice, from 1 (not at all confident) to 10 (extremely confident)	Writing grants and finding funding opportunities<br>Mentoring<br>Being well-versed in your subject matter as a resource for faculty and students alike<br>Leading a lab (project management)<br>Teaching classes<br>Active member of department (academic committee member)<br>Outreach to scientific community (serving on study sections, editorial boards, advisory boards, professional organizations)<br>Communicating scientific findings through talks/conferences and publications<br>Utilizing all available core resources<br>Budgets and financial literacy for large accounts
Common between pre-program and post-program surveys	Please identify how committed you are to pursuing an academic career. The further to the right you select, the more committed you are.	Numerical scale, from 0 to 100	
Pre-program survey	What do you hope to gain from your participation in the program? What are your expectations for growth?	Open end, free text	
Post-program survey	In what ways do you feel you have changed or grown as a result of your participation in the program?		
Post-program survey	Which elements of the program were most valuable to you?	Open end, free text	
Post-program survey	Do you have any suggestions for ways to improve the program in the future?	Open end, free text	
Post-program survey	How strongly do you agree with the following statements:	Single choice, 6 points scale, from (strongly disagree) to (strongly agree)	This program was a valuable experience for me.<br>I would recommend this program to others.

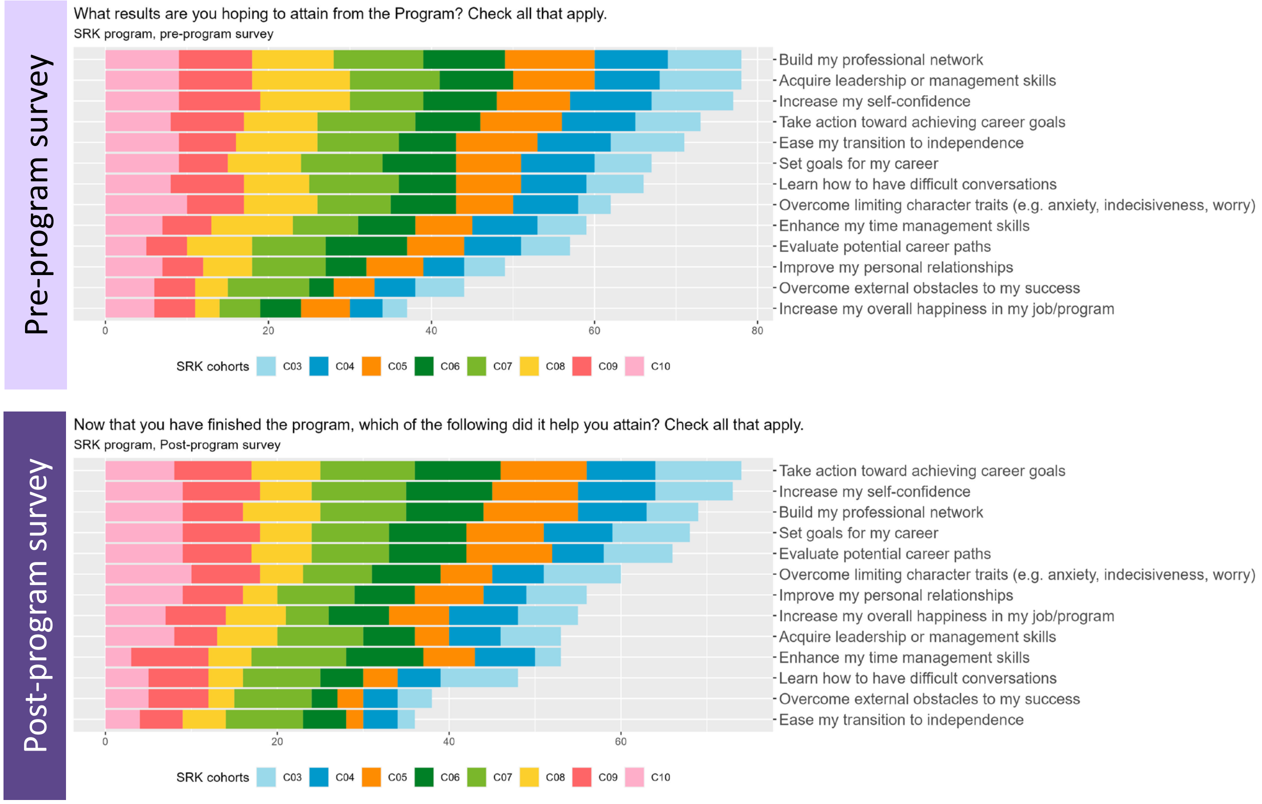

Supplementary Figure 1: The responses of participants from cohort 3 to 10 to the SRK survey questions "What results are you hoping to attain from the Program? Check all that apply." and "Now that you have finished the program, which of the following did it help you attain?" Discussions on these results are included in the main text of the manuscript.

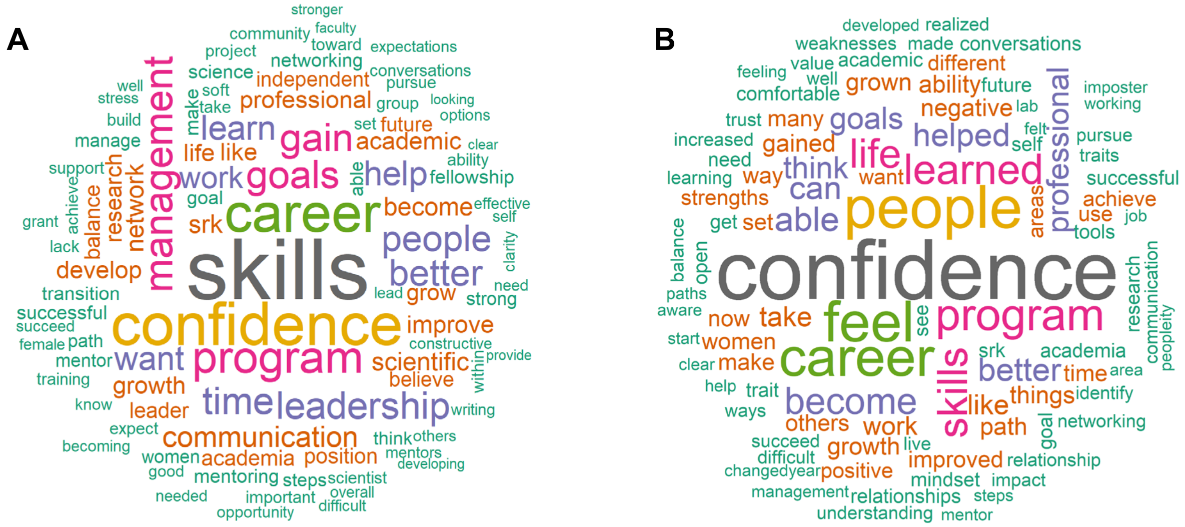

Supplementary Figure 2: Word clouds created from the open-ended responses of participants from cohort 3 to 10 to the SRK survey questions, "What do you hope to gain from your participation in the program?, What are your expectations for growth?" (pre-program) and "In what ways do you feel you have changed or grown as a result of your participation in the program?" (post-program). The size and color of the words corresponds to their frequency to show recurrent topics and keywords mentioned by the program participants.

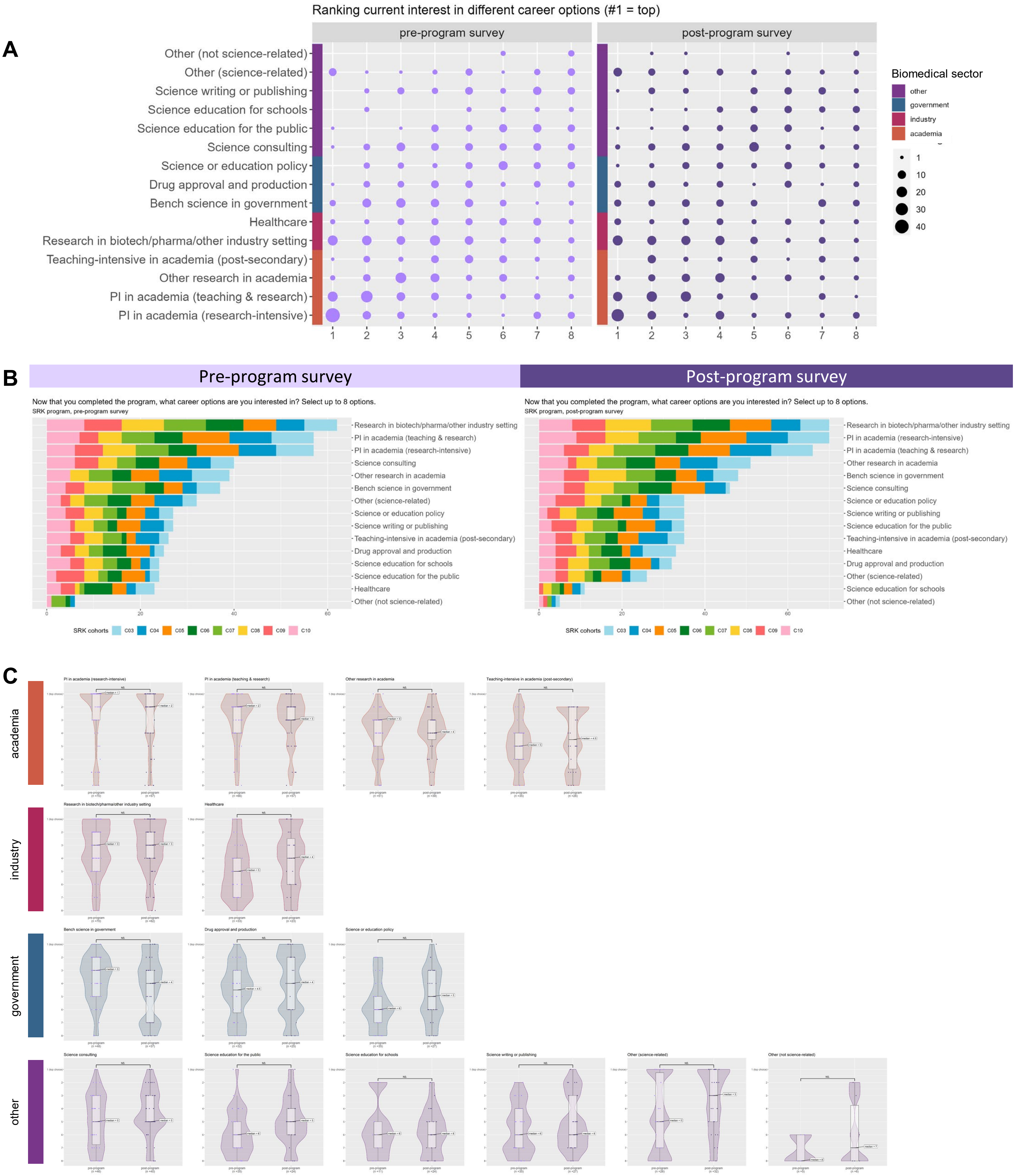

Supplementary Figure 3: Visualization of the responses of participants from cohort 3 to 10 to the SRK survey question "What career options are you currently interested in?" Participants were asked to rank up to 8 of the 15 available options (with rank #1 being the option they are most interested in). Panel A: dotplots where the size of each dot represent the percentage the specific career option was chosen (y-axis) at a specific ranking (x-axis), split between pre-program and post-program survey. Panel B: Pre-program and post-program stacked barcharts accounting for how many times each career option was chosen within the first 8 top ranked. Panel C, the individual ranking and responses are displayed in violin plots arranged into the different high-level biomedical sectors.

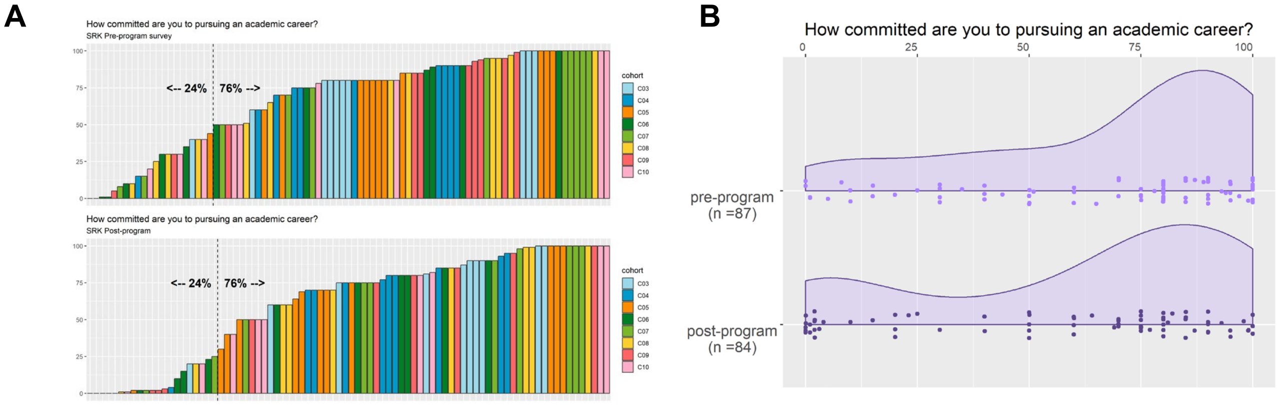

Supplementary Figure 4: The responses of participants from cohort 3 to 10 to the SRK survey question "Please identify how committed you are to pursuing an academic career. The further to the right you select, the more committed you are." Panel A shows a sorted bar char where each bar is an individual response, colored by the cohort the participant belongs to (upper graph: pre-program response, lower graph: post program response). Panel B shows the individual responses as dots on a 0-100 horizontal scale, together with a smoothened distribution line.
No statistical difference was observed between the pre-program and post-program responses.

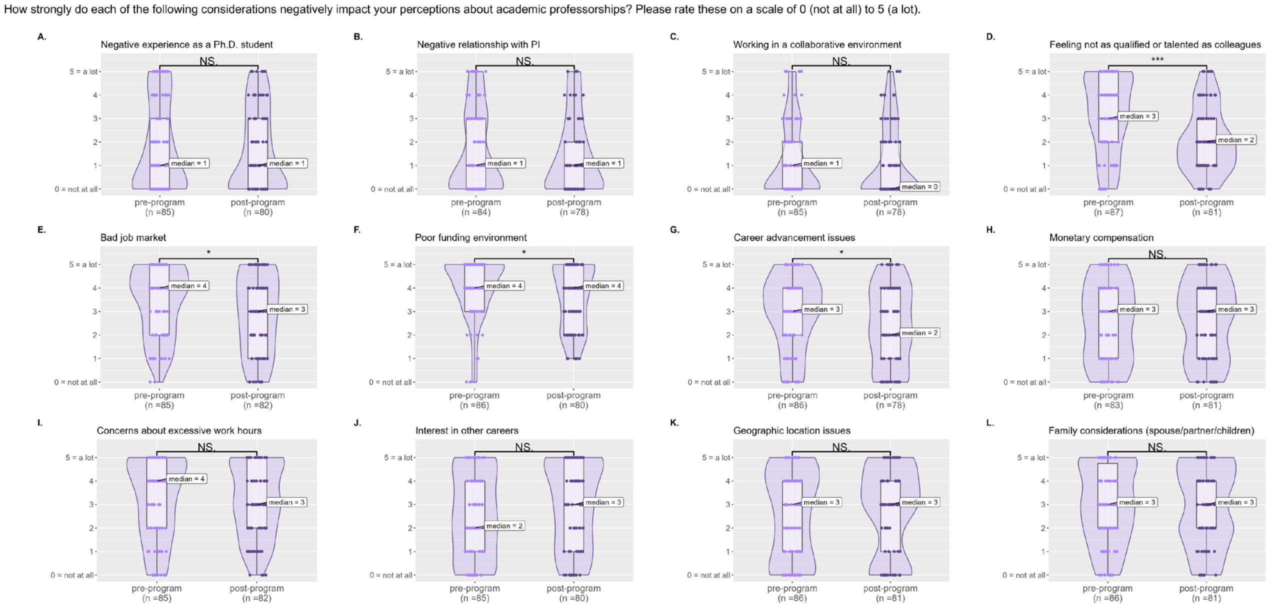

Supplementary Figure 5: The responses of participants from cohort 3 to 10 to the SRK survey question "How strongly do each of the following considerations negatively impact your perceptions about academic professorships? Please rate these on a scale of 0 (not at all) to 5 (a lot)." Each panel shows the pre-program and post-program survey comparison of the ratings. eight out of the twelve responses have non-significant (N.S.) decrease post-program, three out of have modest (* $p < 0.05$) decrease and one "Feeling not as qualified or talented as colleagues"(shown in Panel D) has a strong significant decrease (*** $p <=0.001$).

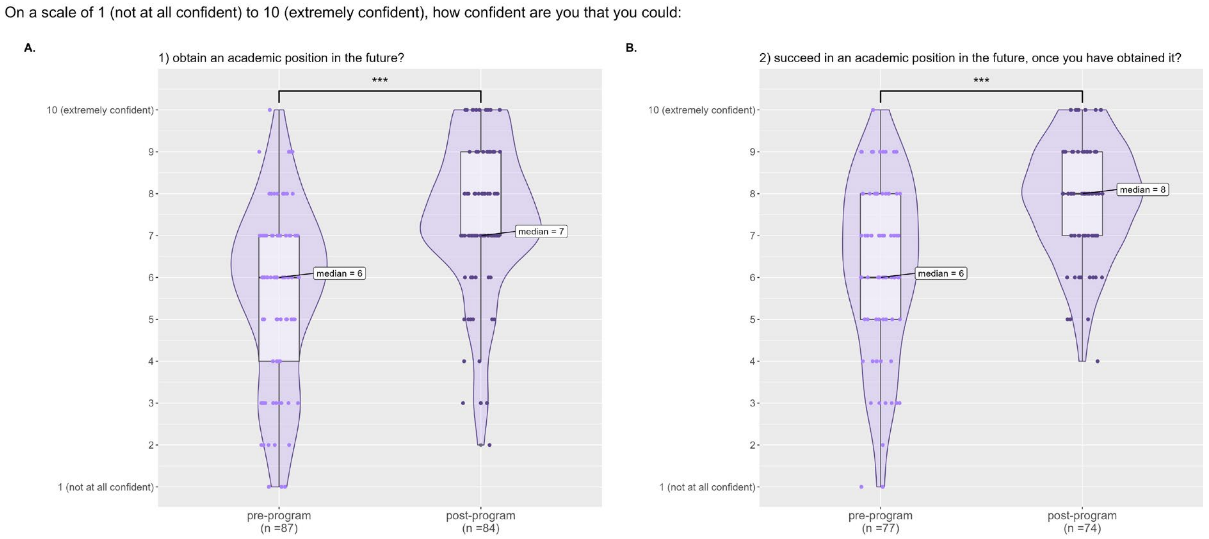

Supplementary Figure 6: The responses of participants from cohort 3 to 10 to the SRK survey question "On a scale of 1 (not at all confident) to 10 (extremely confident), how confident are you that you could: 1) Obtain an academic position in the future? and 2) Succeed in an academic position in the future, once you have obtained it?" Each panel shows the pre-program and post-program survey comparison of the ratings. Both questions have an increase in confidence in post-program responses of strong significance (***: p <=0.001).

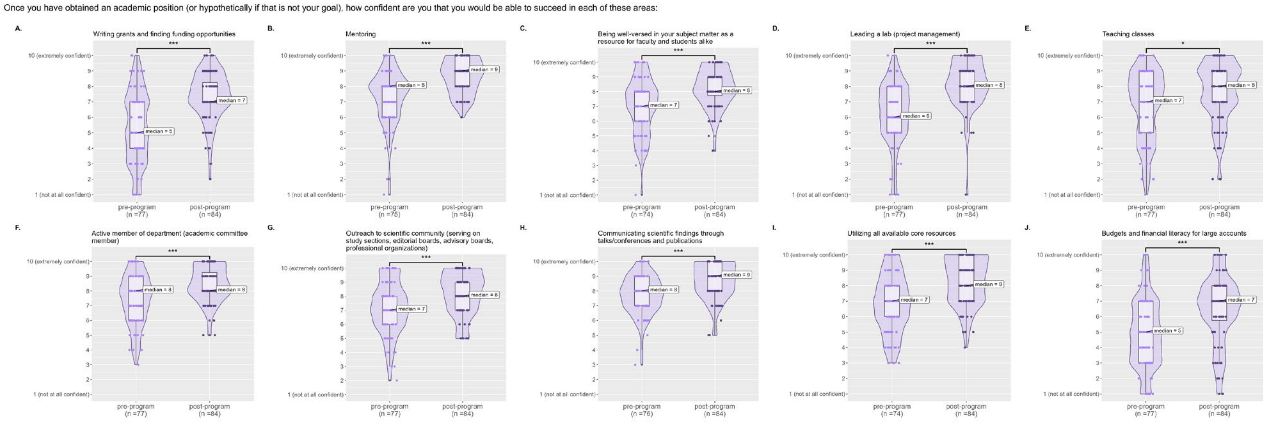

Supplementary Figure 7: The responses of participants from cohort 3 to 10 to the SRK survey question "Once you have obtained an academic position (or hypothetically if that is not your goal), how confident are you that you would be able to succeed in each of these areas." Each panel shows the pre-program and post-program survey comparison of the ratings in each area. Nine of the ten areas show a strongly significant increase in confidence in post-program (marked with ***, p <=0.001) and one area (Panel E) show a small positive increase (p < 0.05)

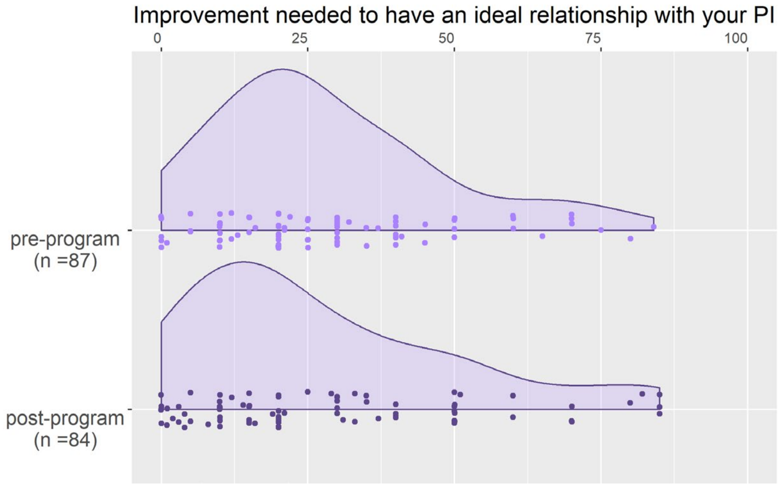

Supplementary Figure 8: The responses of SRK fellows to the survey question “Knowing that no relationship is perfect, how much improvement would be needed for you to have an ideal relationship with your PI? The further to the right you select, the more improvement is needed.” The plots show the individual responses as dots on a 0-100 horizontal scale, together with a smoothened distribution line. No statistical difference was observed between the pre-program and post-program responses.

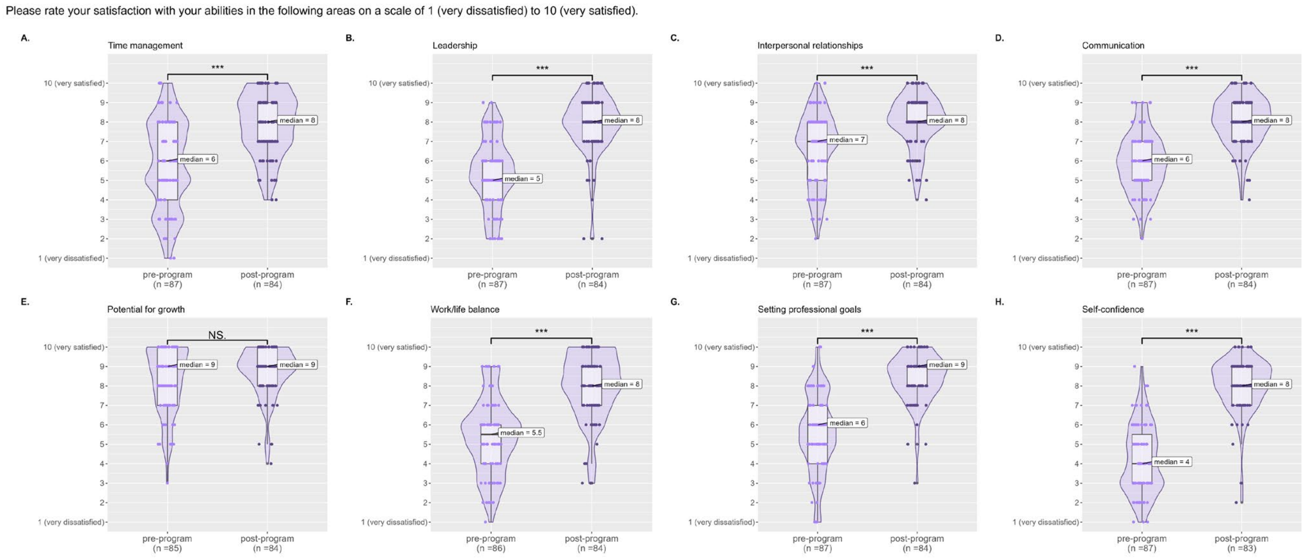

Supplementary Figure 9: The responses of participants from cohort 3 to 10 to the SRK survey question “Please rate your satisfaction with your abilities in the following areas on a scale of 1 (very dissatisfied) to 10 (very satisfied).” Each panel shows the pre-program and post-program survey comparison of the ratings for each ability. Nine of the ten areas show a strongly significant increase in satisfaction in post-program (marked with ***, p <=0.001) and one ability (Potential for growth, Panel E) show a non-significant change, due to the already high satisfaction on that ability in pre-program responses.
